# Stem cell-derived exosomes prevent pyroptosis and repair ischemic muscle injury through a novel exosome/circHIPK3/ FOXO3a pathway

**DOI:** 10.7150/thno.42259

**Published:** 2020-05-18

**Authors:** Bing Yan, Yu Zhang, Chun Liang, Bin Liu, Fengzhi Ding, Yanli Wang, Bao Zhu, Ranzun Zhao, Xi-Yong Yu, Yangxin Li

**Affiliations:** 1 Institute for Cardiovascular Science and Department of Cardiovascular Surgery, First Affiliated Hospital and Medical College of Soochow University, Suzhou, Jiangsu 215123, P. R. China; 2 Department of Cardiology, Changzheng Hospital, Second Military Medical University, Shanghai 200003, P. R. China; 3 Department of Cardiology, the Second Hospital of Jilin University, Changchun, Jilin 130041, P. R. China; 4 The First Affiliated Hospital of Zunyi Medical University, Zunyi, Guizhou, 563000, P. R. China; 5 Key Laboratory of Molecular Target & Clinical Pharmacology and the State Key Laboratory of Respiratory Disease, Guangzhou Medical University, Guangzhou 511436, P. R. China

**Keywords:** circRNA, pyroptosis, exosome, inflammasome, NLRP3

## Abstract

**Rational:** Ischemic injury of the skeletal muscle remains a serious clinical problem and currently there is no effective therapy. The aim of the present study is to determine whether human umbilical cord mesenchymal stem cells- derived exosomes (UMSC-Exo) could repair ischemic injury by releasing circular RNA.

**Methods and Results:** To create hindlimb ischemia, we surgically ligated the left femoral artery in C57BL/6 mice. Using circRNA-seq analyses of total RNA from ischemic and control muscles, we found reduced expression of circHIPK3 in the ischemic muscle. To explore the role of circHIPK3 in ischemic injury, the mice were randomly assigned into three groups after surgery: 1) vehicle; 2) UMSC-Exo; 3) UMSC-Exo and siRNA targeting circHIPK3 (UMSC-Exo /si-circHIPK3). UMSC-Exo treatment significantly increased expression of circHIPK3 and improved blood perfusion, running distance and muscle force, which were reversed by injection of UMSC-Exo /si-circHIPK3, suggesting that UMSC-Exo improve muscle function by releasing circHIPK3. UMSC-Exo treatment also inhibited ischemia induced pyroptosis - cell death caused by inflammasome as evidenced by activation of NLRP3, cleaved caspase-1, and subsequent increase of IL-1β and IL-18, and the effects were reversed by injection UMSC-Exo /si-circHIPK3. Bioinformatic analysis identified miR-421/FOXO3a as a potential target for circHIPK3, which was confirmed by luciferase reporter assay. Knockdown of circHIPK3 in C2C12 cells resulted in increased expression of miR-421. We established an* in vitro* model of pyroptosis by stimulating C2C12 cells with LPS and ATP. LPS and ATP treatment resulted in reduced expression of circHIPK3 and increased expression of miR-421, which was prevented by UMSC-Exo. Western blot analysis showed reduced levels of NLRP3 and cleaved caspase-1 when cells were treated by UMSC-Exo. The expression of FOXO3a in C2C12 cells was increased in the presence of miR-421 inhibitor, and the expression was reduced when cells were treated by LPS and ATP. Importantly, the expression of FOXO3a was upregulated by UMSC-Exo but was reduced when si-circHIPK3 was present.

**Conclusions:** Using loss/gain-of function method, we demonstrated that miR-421/FOXO3a is the direct target of circHIPK3, and UMSC-Exo prevent ischemic injury by releasing circHIPK3, which in turn down regulate miR-421, resulting in increased expression of FOXO3a, leading to inhibition of pyroptosis and release of IL-1β and IL-18.

## Introduction

Acute lower limb ischemia induced injury is a serious condition caused by peripheral artery disease, diabetes, and limb surgeries 1, but the molecular mechanisms underlying the injuries remain unclear. Pyroptosis is a type of programmed cell death resulting from inflammatory assault 2. Unlike apoptosis, pyroptosis is a caspase-1 dependent process that leads to rapid cell lysis and the release of inflammatory content 3. Inflammatory stimuli such as cell debris resulting from ischemic injury can activate inflammasome NLRP3 4, 5. Once activated, NLRP3 triggers pyroptosis by releasing inflammatory cytokines including IL-18 and IL-1β 6, 7. However, the role of this novel signaling pathway in limb ischemia induced injury has not been explored.

Circular RNAs are a novel class of non-coding RNAs that form single stranded, closed loop 8, 9. Our novel circRNA/mRNA sequencing data from normal and hindlimb ischemia tissues indicates that circHIPK3 was one of the most abundant circRNA in muscle and its expression was dramatically decreased under ischemic condition. It is known that circHIPK3 regulates cell growth 10. The circRNA/mRNA sequencing data also revealed changes of gene expression that is consistent with the activation of inflammasome cascade in ischemic muscles. Bioinformatic analysis suggests miR-421/FOXO3a as a potential signaling pathway downstream of circHIPK3. FOXO3a is a transcription factor of the O subclass of the forkhead family that inhibits pyroptosis by regulating inflammatory response 11, 12. However, the role of circHIPK3/miR-421/FOXO3a in hindlimb ischemic injury remains undefined.

Exosomes are microvesicles that contain noncoding RNAs such as miRNA and circRNA 13, 14, and are secreted by many different types of cells. We and others have demonstrated that stem cell-derived exosomes can be used as carrier to deliver miRNA to repair ischemic tissues 15-19 . However, it is unknown whether exosomes derived from human umbilical cord mesenchymal stem cells (UMSC-Exo) could deliver circHIPK3 to repair hindlimb ischemic injury.

Using circRNA/mRNA sequencing data and bioinformatics analysis as guidance, and loss-of-function and gain-of-function approaches, we identified the role of circHIPK3/miR-421/FOXO3a signaling pathway in hindlimb ischemic injury. Moreover, we investigated whether circHIPK3 played a role in mediating UMSC-Exo based therapy for tissue repair, and in preventing inflammasome-induced pyroptosis.

## Methods

### Harvest and identification of UMSC exosomes

Human umbilical mesenchymal stem cells (UMSCs; Jiangsu Heze Biotechnology Co., Ltd., China) were cultured in minimum essential medium (MEM) with 10% fetal bovine serum (FBS), The FBS had been centrifuged at 100,000 g to eliminate preexisting bovine-derived exosomes. After 48 h in culture, exosomes were isolated from UMSC culture supernatant using a total exosome isolation kit (Life Technology, Grand Island, NY, USA). The culture medium collected from UMSCs was centrifuged at 2,000 g for 30 min to remove dead cells and debris, and then transferred to a new tube containing 0.5 volumes of the Total Exosome Isolation reagent. The mixture was incubated at 4°C overnight and centrifuged at 10,000 g for 1 h at 4°C. The pellets (exosomes) were re-suspended in phosphate buffer saline. The concentration of exosomes was determined using a bicinchoninic acid (BCA) protein assay kit (Takara, Japan). The exosomes were attached to aldehyde/ sulphate latex beads (4 μm, Molecular Probes, Invitrogen), then incubated with a FITC‐conjugated antibody against CD63 (Abcam), and the expression of CD63 was analysed by flow cytometry. In separate experiments, the expression of exosome marker CD63, CD9, TSG101 was also analyzed by Western blot.

### Exosome labeling with PKH26

Purified exosomes were labeled with PKH26 fluorescent labeling kit (Sigma Aldrich, USA). C2C12 cells were grown to 50% confluence in 12-well plates, and then the medium was replaced with DMEM containing PKH26-labeled exosomes. After incubation for 24 h at 37°C in 5% CO2 atmosphere, the cells were washed twice with PBS, fixed, and nuclei were stained with 4′, 6-diamidino-2-phenylindole (DAPI). Finally, the sample of the cells was determined with fluorescence microscopy.

### Cell culture and loss/gain approach

The murine myoblast line (C2C12) cells were cultured in Dulbecco's modified Eagle's medium (DMEM) supplemented with 10% FBS in 5% CO_2_ at 37℃_._ To induce differentiation, medium was switched to DMEM containing 2% horse serum (Gibco, Cat# 1852632) when the density of cells reached 70-80% and cultured for 6 days. In order to induce the formation of inflammasome, C2C12 cells were cultured in medium containing Escherichia coli 0111:B4 LPS (100 ng/mL; Sigma-Aldrich) for 2 h, followed by adding ATP (2.5 mM; Sigma-Aldrich) and cultured for 1 h 20.

MiR-421 mimic, miR-421 inhibitor and small interference RNA (si-circHIPK3) targeting circHIPK3 were synthesized by Ribobio (Guangzhou, China). The cells were transfected using Lipofectamine 2000 (Invitrogen, Carlsbad, CA, USA) according to the manufacturer's instructions. For experiments involving Exo- si-circHIPK3, the cells were transfected with small interference RNA (si-circHIPK3) targeting circHIPK3 (GGTACTACAGGTATGGCCT) for 48 hours, then the exosomes were isolated as mentioned above.

### EdU proliferation assay

Cell proliferation was assessed using EdU Cell Proliferation Assay kit (RiboBio, Guangzhou, China). After different treatments, C2C12 cells were incubated in fresh medium containing 10 μM EdU for 24 h, then C2C12 cells were washed with PBS, and fixed with 4% paraformaldehyde for 30 min, treated with 0.5% Triton X-100 for 10 min. The cell nuclei were stained with Hoechst 3342 for 15 minutes. Finally, the proportion of the cells incorporating EdU was determined with fluorescence microscopy.

### Enzyme-linked immunosorbent assay (ELISA)

Serum IL-1β and IL-18 concentrations were measured using an ELISA kit according to manufacturer's instructions. IL-1β ELISA kit was from RayBiotech, Inc., Norcross, GA, USA, IL-18 ELISA kit (Cat# EK218) was from MultiSciences Biotech Co. (Hangzhou, China)

### Dual-luciferase reporter assay

The 3'-UTR of FOXO3a and circHIPK3 containing the conserved miR-421 binding sites and was amplified using PCR. The recombinant luciferase reporter plasmids contained the potential miR-421 binding site sequences of the FOXO3a and circHIPK3. HEK293T cells were seeded in 96-well plates (5 ×10^3^ cells per well) and cultured for 24 h prior to transfection. The cells were co-transfected with a mixture of 50 ng reporter plasmid (psiCHECK2 -3'UTR -circHIPK3 or psiCHECK2 -3'UTR FOXO3a vector), with 200 nM miR-421 mimic from RiboBio. After 48 h, the luciferase activity was analyzed using a dual luciferase reporter assay system (Promega, USA) per manufacturer's instructions.

### RNA extraction and real-time PCR

RNA was extracted using Trizol reagent (TaKaRa Biotech, Japan), and cDNA was synthesized using Prime Script™ RT reagent (TaKaRa Biotech). The relative expression levels of target genes were determined using SYBR^®^ Premix Ex Taq™ (TliRNaseH Plus) (TaKaRa Biotech). Real-time PCR was performed using the ABI (Foster City, CA, USA) StepOnePlus Real-Time PCR System with the following primers:

Human circHIPK3: forward, 5′-TATGTTGGTGGATCCTGTTCGGCA-3′, reverse, 5′-TGGTGGGTAGACCAAGACTTGTGA-3′;

Human GAPDH: forward, 5′-GGTGGTCTCCTCTGACTTCAACA-3′, reverse, 5′-GTTGCTGTAGCCAAATTCGTTGT-3′;

Mouse circHIPK3: forward, 5'-GGATCGGCCAGTCATGTATC-3', reverse, 5'-ACCGCTTGGCTCTACTTTGA-3';

Mouse GAPDH: forward, 5′-AAATGGTGAAGGTCGGTGTG-3′, reverse, 5′-TGAAGGGGTCGTTGATGG-3′;

Mouse FOXO3a: forward, 5′-AAACGGCTCACTTTGTCCCA-3′, reverse, 5′-TTGTGCCGGATGGAGTTCTTC-3′;

Mouse NLRP3: forward, 5′-CACTCATGTTGCCTGTTCTTC-3′′, reverse, 5′-CGGTTGGTGCTTAGACTTGA-3′;

Mouse IL-1β: forward, 5′-CAACCAACAAGTGATATTCTCCATG-3′, reverse, 5′-GATCCACACTCTCCAGCTGCA-3′;

Mouse IL-18: forward, 5′-ACGTGTTCCAGGACACAACA-3′, reverse, 5′-GGCGCATGTGTGCTAATCAT-3′.

The miR-421 and U6 primers were from RiboBio. Relative expression was calculated from cycle threshold (Ct) using the following equation: relative expression = 2^-(SΔCt-CΔCt)^. GAPDH and U6 were used as the internal reference.

### Western blot

Total proteins extracted by RIPA buffer (Beyotime Biotechnology, China) were separated by SDS-polyacrylamide gel electrophoresis (SDS-PAGE), and then transferred to polyvinylidene fluoride (PVDF) membranes (Millipore ISEQ00010). The membranes were first incubated with primary antibodies followed by secondary antibodies. The primary antibodies against NLRP3 (Cat# ab214185) and Caspase-1(Cat# ab1872), were from Abcam (Cambridge, UK). Antibodies against GAPDH (Cat#Mab5465-100) and HRP-linked anti-rabbit Immunoglobulin G (IgG) (Cat#GAR007) were from MultiSciences Biotech Co. (Hangzhou, China). Antibody against FOXO3a (Cat#D19A7) was from Cell Signaling Technology (Danvers, MA, USA). The primary antibodies against CD63 (catalog # ab134045), CD9 (catalog # ab92726), and TSG101 (catalog # ab125011) were from Abcam (Cambridge, MA, USA). Another primary antibody against CD63 (catalog # sc-5275) was from Santa Cruz Biotechnology (Dallas, TX, USA). The protein signals were detected using an ECL chemiluminescence kit (Biological Industries) and the luminescence was visualized using a BioRad luminescent imaging system.

### Mouse unilateral hindlimb ischemia model

Adult male 8-12-week-old C57BL/6 mice were supplied by the Experimental Animal Center of Soochow University (Suzhou, China). The animal experiments were approved by the Animal Care and Use Committee of Soochow University. The mice were randomly assigned into three treatment groups, PBS, exosomes, exosomes- si-circHIPK3. Unilateral mouse hindlimb ischemia was created by ligating the left femoral artery under general anesthesia (2-4% isoflurane in oxygen). Immediately after surgery, the muscle of the ischemic hindlimb was injected with one of the above mentioned treatments, including that 100 μg exosomes were injected into the muscle of each mouse at three injection points.

### Laser Doppler perfusion imaging of mouse hindlimb

Laser Doppler perfusion imager (LDPI, Moor Instruments, Axminster, UK) was used to monitor blood flow in mice hindlimb at 1,3,7,14,21 and 28 days after the surgery. Perfusion was expressed as the ratio of the ischemic over the contralateral, non-manipulated leg.

### Running endurance

On the 28th day post surgery, each group of mice was exercised following a run-to-exhaustion protocol. Prior to running, mice were acclimated to the treadmill (Jiangsu SANS Biological Technology Co. Ltd.) for 1-2 h and to the motor sound for 15 min. The belt was initially set at a slow speed (6 m/min), then the velocity was increased 2 m every 2 min for the first 12 min and held steady (18 m/min). Exhaustion was defined as the point when mice spent more than 10 consecutive seconds on the shock grid without seeking to re-engage the treadmill.

### Muscle force measurement

At day 28 after the surgery, mouse grip strength was measured using a Grip Strength Meter (Ji-Nan Biotechnology, Shandong, China) for 3 consecutive days. For each day, 6 grip strengths were assessed at 1-min intervals, and the average grip strength over 3 days was calculated.

### circRNA/mRNA sequencing and analysis

Male 8-week-old C57BL/6 mice were randomly assigned into two groups, control (n = 3) and ischemia group (n = 3). Unilateral mouse hindlimb ischemia group was created by ligating the left femoral artery under general anesthesia (2-4% isoflurane in oxygen). We extracted gastrocnemius muscle tissue of the ischemic hindlimb and control group. Total RNA was extracted by Trizol reagent (Invitrogen), ribosome depleted RNA samples were fragmented and then used for first- and second-strand complementary DNA (cDNA) synthesis with random hexamer primers. Whole transcriptome sequencing data obtained from HiseqTM Sequencer was filtered (removing the adaptor sequences, reads with > 5% ambiguous bases (noted as N) and low-quality reads containing more than 20 percent of bases with qualities of < 20) and mapped to mouse genome utilizing HISAT2. HTSeq was used to calculate the gene count of mRNA and circRNA. All RNA-seq and bioinformatic analysis were performed at NovelBio Ltd (Shanghai, China).

### Proteomics sequencing sample preparation

The tissues were grounded in liquid nitrogen, lysed by adding lysis buffer (7M urea, 4% SDS, 1× Protease Inhibitor Cocktail (Roche Ltd. Basel, Switzerland)), followed by sonication on ice. The samples were centrifuged at 13, 000 rpm for 10 min at 4℃ and supernatant was collected. The protein concentration was analyzed using the BCA protein assay, and 100 μg protein was transferred into a new tube and the final volume was adjusted to 100 μL with100 mM TEAB (triethylammonium bicarbonate). The samples were incubated with 5 μL DTT (200 mM) at 55℃ for 1 hour, then 5 μL of the 375 mM iodoacetamide solution was added and incubated for 30 minutes in order to prevent the re-formation of the disulfide bond. The proteins were precipitated with ice-cold acetone, then re-dissolved in 100 μL TEAB. Proteins were digested with sequence-grade modified trypsin (Promega, Madison, WI), and the digested peptide mixture was labeled using chemicals from the iTRAQ reagent kit. The labeled samples were combined, desalted using C18 SPE column (Sep-Pak C18, Waters, Milford, MA) and dried in vacuum.

### High pH reverse phase separation

The peptide mixture was dissolved in buffer A (buffer A: 10 mM ammonium formate in water, pH 10.0, adjusted with ammonium hydroxide), and then fractionated by a linear gradient high pH separation using an Aquity UPLC system (Waters Corporation, Milford, MA). The column flow rate was maintained at 250 μL/min and column temperature was maintained at 45℃. Twelve fractions were collected, and each fraction was dried in a vacuum concentrator for the next step.

### Low pH nano-HPLC-MS/MS analysis

The fractions were resuspended with 40 μL solvent C (water with 0.1% formic acid; D: ACN with 0.1% formic acid), separated by nanoLC and analyzed by on-line electrospray tandem mass spectrometry. The experiments were performed on an EASY-nLC 1000 system (Thermo Fisher Scientific, Waltham, MA) connected to an Orbitrap Fusion Mass Spectrometer (Thermo Fisher Scientific, San Jose, CA) equipped with an online nano-electrospray ion source. The samples (4 μL) were loaded onto the trap column, with a flow rate of 10 μL/min for 3 min and subsequently separated on the analytical column with a linear gradient, from 5% D to 30% D in 110 min.

### Quantitative data analysis

All Proteomics sequencing and related Bioinformatics analysis were performed at Biotree Biotech Co.Ltd. (Shanghai, China). Briefly, the percolator algorithm was used to control peptide level false discovery rates (FDR) lower than 1%. Only unique peptides were used for protein quantification. Protein contains at least two unique peptides, and the method of normalization on protein median was used to correct experimental bias, the minimum number of proteins that can be observed was set to 1000.

### Statistical analysis

Data were presented as mean ± SD. Multiple comparisons were analyzed by ANOVA with post-hoc analysis by the Newman-Keuls test. Two-tailed t-tests were used to determine the significance of differences between two groups. P < 0.05 was considered statistically significant.

## Results

### circRNA/mRNA-seq analysis of circRNAs profile in muscle tissue

**Figure 1:**
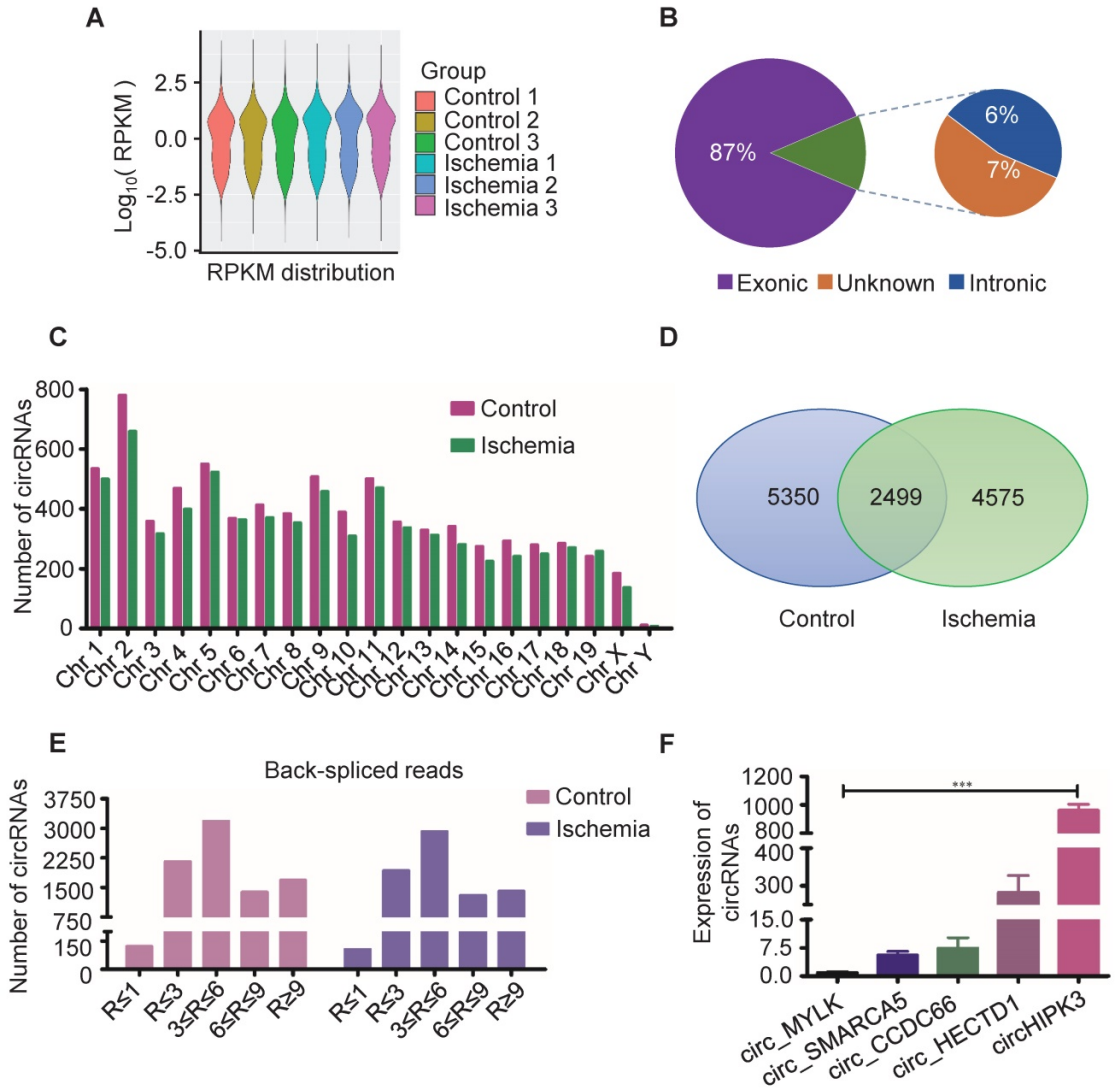
**Profile of circRNAs in ischemia and control muscle. (A)** RNA-seq analyses of circRNA from ischemic and normal muscles. RPKM distribution showed good repeat among 3 samples. **(B)** Reads distribution of circRNA in genome. The circRNAs identified from exonic, intronic, and unknown were shown in “Purple”, “Blue” and “Brown”, respectively.** (C)** The quantity of circRNAs derived from different chromosomes. The circRNAs identified in control and ischemic samples are shown in “Red” and “Green”, respectively. **(D)** Venn diagram of circRNAs expression in ischemia and control groups. **(E)** circRNA-seq analyses showed the Read number in skeletal muscle. Read > 9 indicated higher expression. **(F)** RT-PCR analysis of some circRNA expression in normal mouse muscle.

Circular RNAs are a novel class of non-coding RNAs that have been implicated in acute myocardial infarction 21-23, and in acute lower limb ischemic injury, but the underlying mechanisms are not fully understood. In order to further explore the pathogenesis of acute lower limb ischemic disease, and promote ischemic tissue repair, we characterized circRNA transcripts using RNA sequencing (RNA-seq) analyses of ribosomal RNA-depleted total RNA from ischemia muscle tissue and normal muscle tissue. The data showed good repeat among 3 samples (Figure 1A), 87% of circRNAs were from protein coding exons, 6% of circRNAs were from intronic, 7% of circRNAs were from unknown region (Figure 1B), and number of circRNAs are widely distributed uniformly on the chromosomes (Figure 1C). 7074 circRNAs were detected in ischemia groups, and 7849 circRNAs in the control groups (Figure 1D). 1404 circRNAs were highly expressed in ischemia groups (Read > 9), and 1683 circRNAs were highly expressed in the control groups (Figure 1E). One of these circRNAs (circHIPK3), derived from the HIPK3 gene Exon 2, is highly conservative between humans and mice, and has higher expression than others as determined by circRNA sequencing. Moreover, circRNAs (circ_MYLK, circ_SMARCA5, circ_CCDC6, circ_HECTD1) that promote cell proliferation and inhibit apoptosis can be detected by RT-PCR in normal muscle. Furthermore, the expression of circHIPK3 is much higher compared to that of circ_MYLK, circ_SMARCA5, circ_CCDC6, circ_HECTD1. These data are consistent with the results of RNA-seq (Figure 1F). The data indicate that circHIPK3 may play a significant role in the development of acute ischemia- induced skeletal muscle injury.

### Exosomes promote ischemic muscle repair by delivering circHIPK3* in vivo*

**Figure 2:**
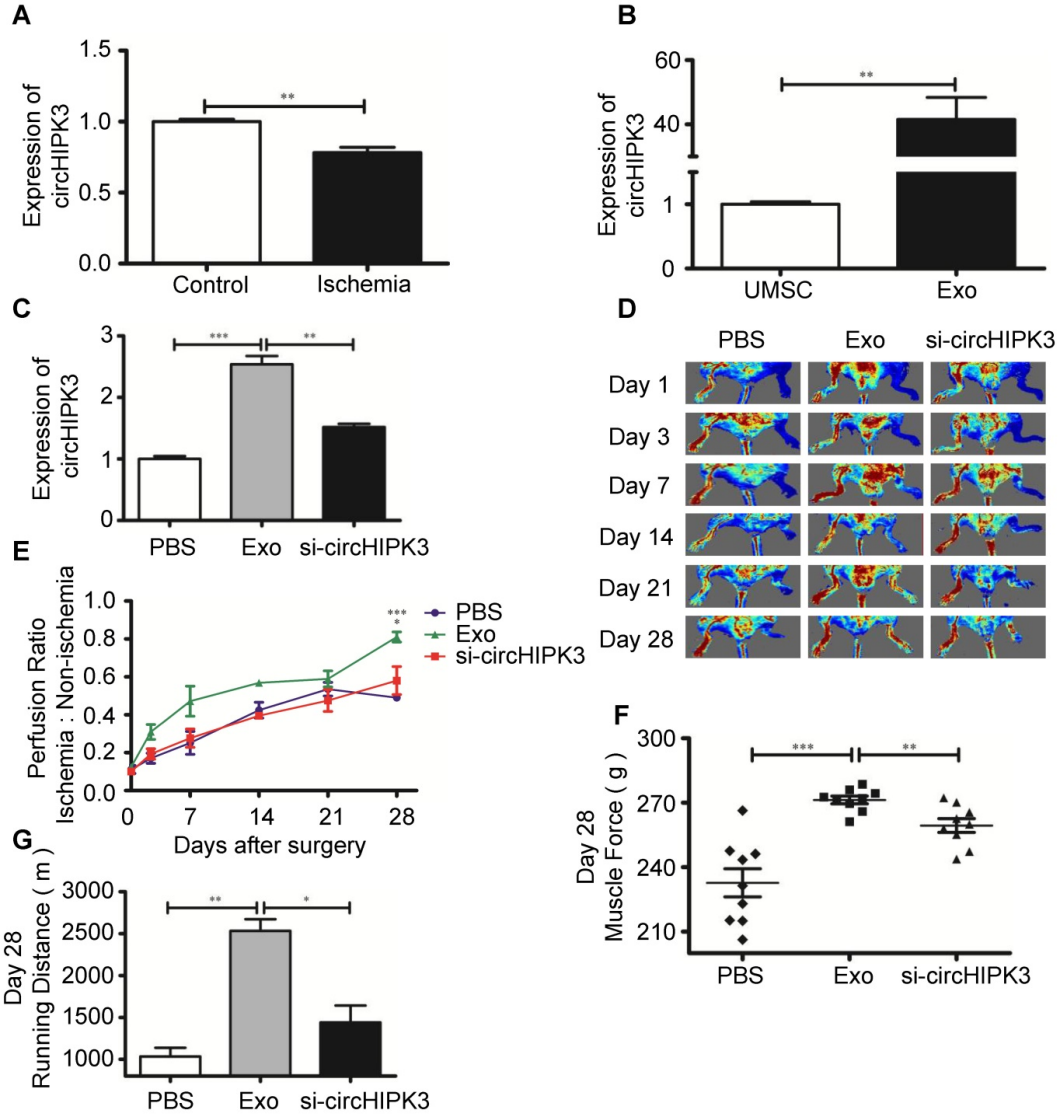
**UMSC-Exo enhances ischemic muscle repair by up regulating circHIPK3. (A)** RT-PCR analysis of circHIPK3 expression in ischemic muscle and control. **(B)** RT-PCR analysis of circHIPK3 expression in UMSC and UMSC-Exo. **(C)** RT-PCR analysis of circHIPK3 expression in ischemic muscle that were treated with PBS, UMSC-Exo, or Exo-si-circHIPK3.** (D)** Laser Doppler perfusion imaging of limbs from mice in different treatment groups after ischemic surgery. **(E)** Blood flow recovery after ischemic surgery.** (F)** Muscle force. **(G)** Running distance. Data is shown as mean ± standard deviation (n=3). *P <0.05, **P <0.01, ***P <0.001.

Exosomes contain functional miRNAs and circRNAs. MiRNAs play an important role in ischemic tissue repair, however, the role of circRNAs in muscle repair has not been explored 24, 25. A recent study showed that circHIPK3 promotes angiogenesis and endothelial cell proliferation 8. We sought to determine whether circRNA is involved in repairing ischemic hindlimb. Real-time PCR revealed that the expression of circHIPK3 was significantly reduced in ischemia hindlimb (Figure 2A), but was highly expression in UMSC-Exo (Figure S2, Figure 2B). To determine whether exosomes can be used to repair ischemic muscle and whether circHIPK3 could be a mediator of exosomes potential beneficial effect, we used siRNA to knockdown circHIPK3 expression in exosome (Figure S3), and performed the following treatment to ischemic hindlimb: (1) vehicle (PBS); (2) Exo (100 µg); (3) Exo-si-circHIPK3 (100 µg). The expression of circHIPK3 was significantly upregulated 3 days after exosome treatment (Figure 2C). Perfusion was quantified 24 h after treatment and up to 28 days thereafter by Laser Doppler imaging, and the results showed that exosome treatment significantly enhanced perfusion at days 7, 14, 21, and 28 (Figure 2D-E). Furthermore, exosome treatment also improved muscle force and the running distance (Figure 2F-G). However, these beneficial effects exerted by exosomes were not observed in Exo-si-circHIPK3, suggesting that UMSC-Exo promotes ischemic hindlimb repair by releasing circHIPK3.

### Inflammatory reactions erupt in ischemic muscle

**Figure 3:**
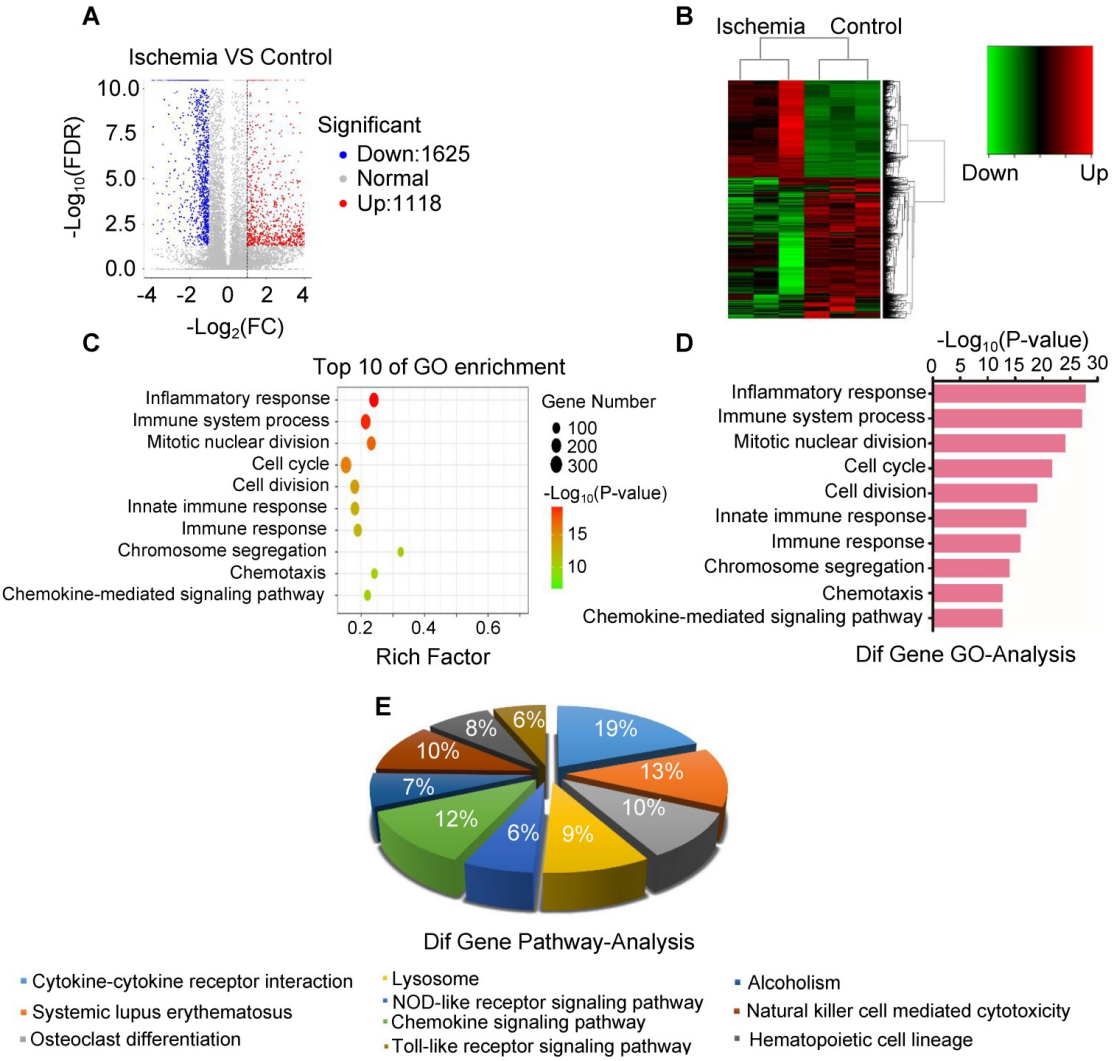
**Analysis of the expression of mRNA and associated pathway. (A)** Volcano map of mRNA sequencing data from ischemic and control groups (blue, downregulated; red, upregulated). **(B)** Heatmap of mRNA sequencing data from ischemic and control groups (green, downregulated; red, upregulated). **(C, D)** Gene ontology (GO) in ischemic muscle compared with control group. **(E)** Analysis of top 10 pathways in ischemic muscle by mRNA sequencing.

To elucidate the key molecule and potential signaling pathways involved in acute skeletal muscle ischemic injury, we analyzed mRNA transcripts using RNA sequencing (RNA-seq) analyses of ribosomal RNA-depleted total RNA from ischemic and normal gastrocnemius muscles. A total of 16300 distinct mRNA candidates were found in the muscles. A total of 2743 mRNAs were found differentially expressed between ischemic and normal muscles, of which 1625 were downregulated and 1118 were upregulated (Figure 3A-B). Differentially expressed mRNAs were also defined by using a FDR (False Discovery Rate) threshold and log_2_ FC (fold-change) analysis through EBSeq algorithm. The threshold of truly significant mRNA was defined as FDR < 0.05 and log_2_ FC > 1 or< -1. The predicted targets of the differentially expressed mRNAs were then analyzed in terms of their gene ontology (GO) categories and pathways using Fisher's exact test and χ^2^ test. Pathway annotations of genes were predicted from KEGG (http://www.genome.jp/kegg/). In the biological process, we found several pathways, including GO: 0006954 (Inflammatory response pathway) and GO: 0002376 (immune system process) (Figure 3C-D), which were significantly upregulated in ischemic muscle. KEGG pathway analysis also revealed that PATH: 04621 (NOD-like receptor signaling pathway) and PATH: 04620 (Toll-like receptor signaling pathway) are significantly activated (Figure 3E). These findings led us to focus on inflammatory response pathway and related molecules.

**Figure 4:**
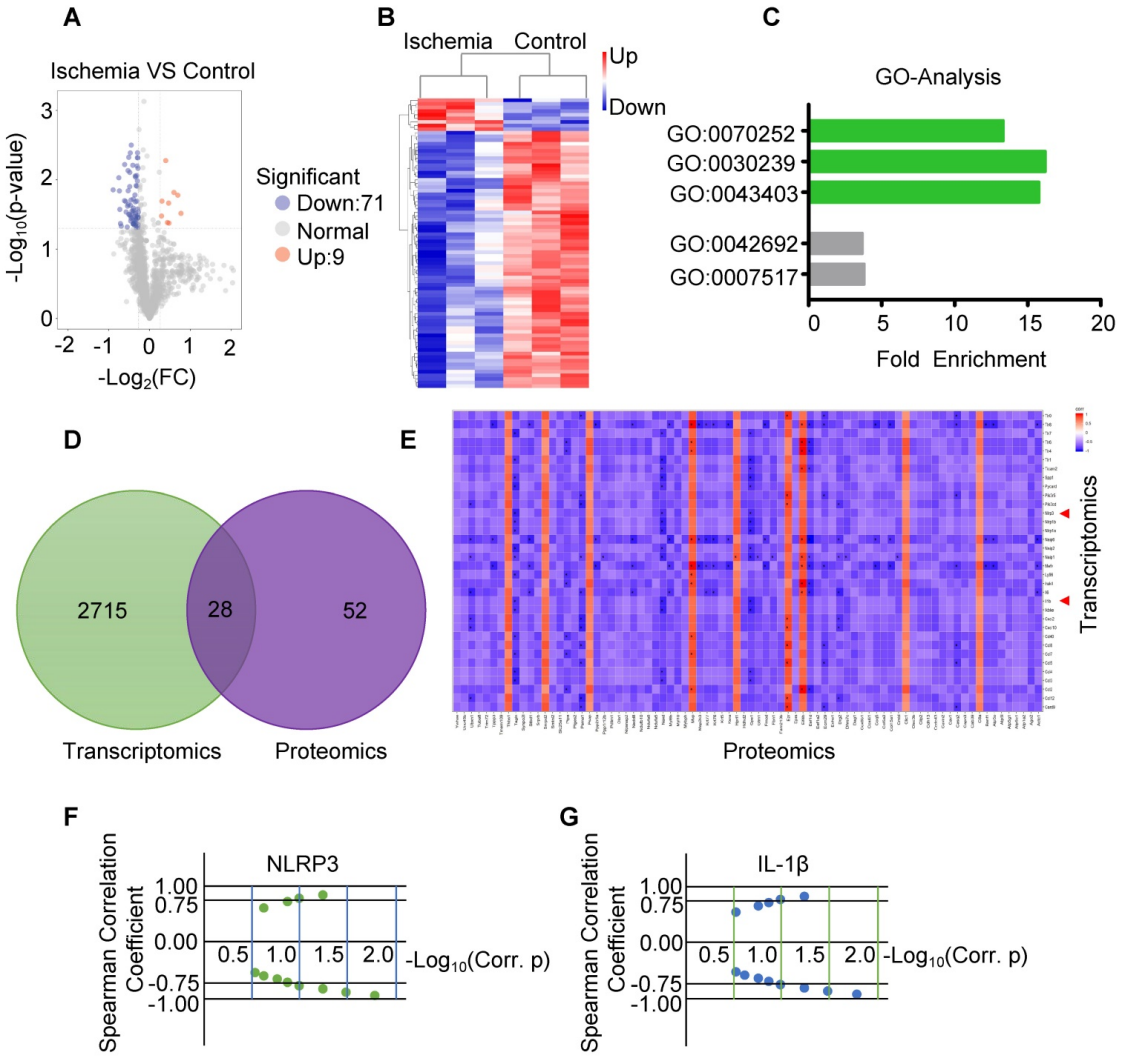
**Analysis of the expression of protein by protein-sequencing. (A)** Volcano map of protein sequencing data from ischemic and control muscles (blue, downregulated; red, upregulated). Significant differences (P-Value < 0.05 and Fold Change >1.2 or Fold Change < 0.83) comparing ischemia to control group**. (B)** Hierarchical cluster data from ischemia and control (blue, downregulated; red, upregulated). **(C)** Gene ontology (GO) analysis of the increased biological process in ischemic muscle.** (D)** Venn diagram of the expression of mRNAs and proteins in ischemia and control muscle. **(E)** The spearman's correlation coefficients using normalized scores (z-scores) to calculate the protein-mRNA correlation. **(F, G)** Spearman's correlation showed the increased expression of NLRP3 and IL-1β mRNA correlation with the increased expression of proteins in ischemia compared to control groups.

To determine the correlation between RNA and protein, part of the same samples for RNA sequencing were used for protein sequencing. Volcano map and hierarchical cluster revealed 80 differentially expressed proteins with statistical significance (P-Value< 0.05 and Fold Change > 1.2 or Fold change< 0.83) between ischemia and control (Figure 4A-B)**.** GO analysis of the sequencing data obtained from ischemic muscles showed enrichment of 3 specific biological processes including GO:0070252 (actin-mediated cell contraction), GO:0030239 (myofibril assembly) and GO:0043403 (skeletal muscle tissue regeneration) (Figure 4C). Venn diagram revealed that 28 genes and related proteins had similar change based on transcriptomic and proteomic analysis in ischemia and control groups (Figure 4D). Heat map analysis revealed similar correlation of these genes and related proteins between ischemic muscle and control muscle (Figure 4E). Importantly, the protein-mRNA correlation of NLRP3 and IL-1β was calculated via spearman's correlation coefficients using normalized scores, and both mRNA and protein were significantly increased in ischemic muscle compared to control (Figure 4F-G).

### circHIPK3 inhibits skeletal muscle pyroptosis *in vivo*

**Figure 5:**
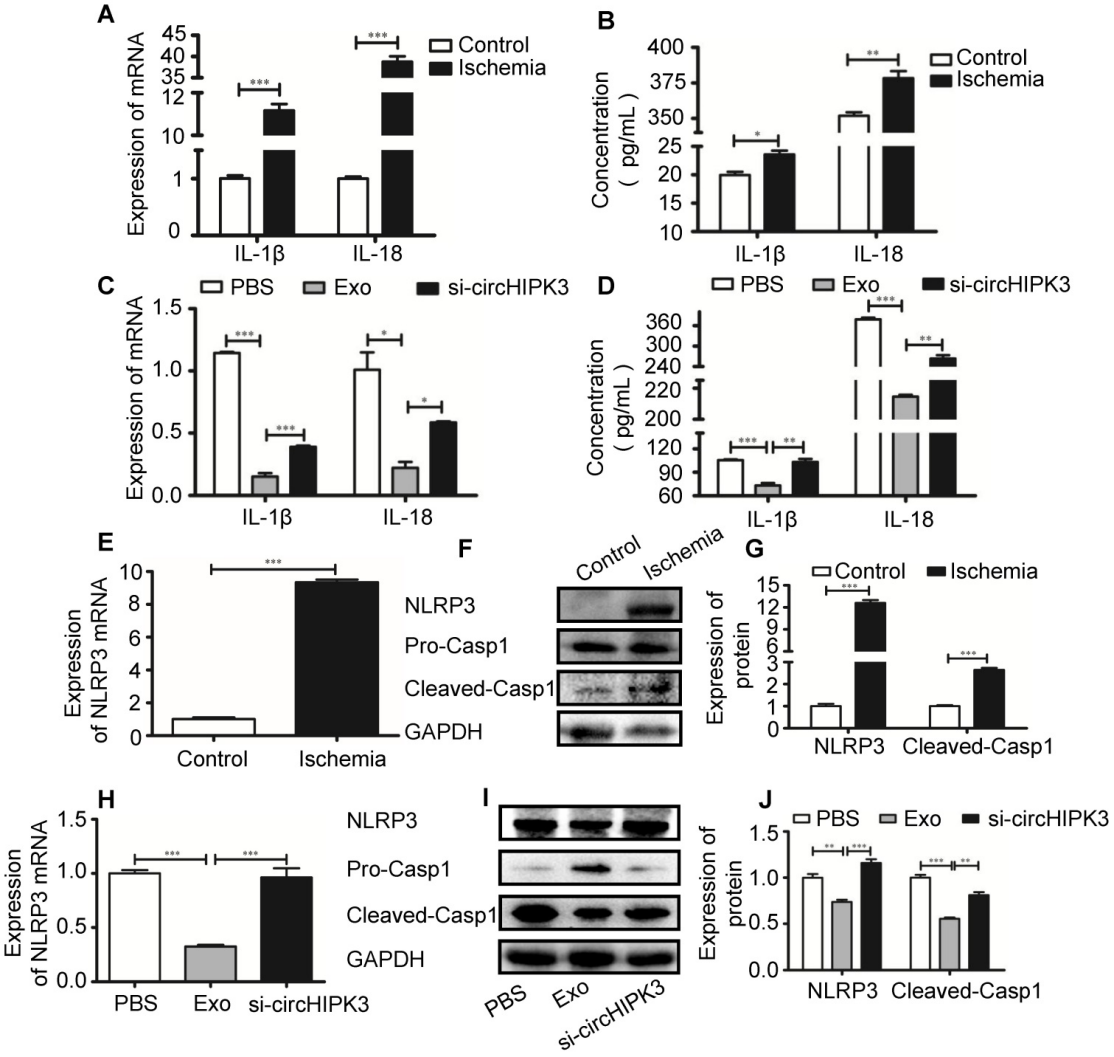
**circHIPK3 from UMSC-EXo inhibits inflammasome induced pyroptosis in skeletal muscle. (A)** RT-PCR analysis of IL-1β and IL-18 mRNA expression in control and ischemic muscle. (**B)** ELISA analysis of serum IL-1β and IL-18 levels from control and ischemia groups.** (C)** RT-PCR analysis of IL-1β and IL-18 mRNA expression in PBS, Exo, and Exo-si-circHIPK3 groups.** (D**) ELISA analysis of serum IL-1β and IL-18 levels in the PBS, Exo, and Exo-si-circHIPK3 groups. (**E)** RT-PCR analysis showed increased NLRP3 mRNA expression in muscle after ischemic surgery. (**F, G)** Western blot analysis showed increased NLRP3 and Casp1 protein expression in muscle after ischemic surgery. **(H)** The mRNA expression of NLRP3 in muscle was decreased in the treatment of the Exo.** (I, J)** Expression of NLRP3 and Casp1 proteins in the PBS, Exo and Exo- si-circHIPK3 treated muscles. Data is shown as mean ± standard deviation (n=3). *P <0.05, **P <0.01, ***P <0.001.

Recent studies showed that pro-inflammatory cytokines such as IL-1β are released in acute myocardial infarction 20, 26. CircVMA21 was able to alleviate inflammatory cytokines-induced NP cell apoptosis through miR-200c-XIAP pathway 27. To determine whether circHIPK3 plays a role in regulating the expression of inflammatory cytokines, we measured the mRNA and protein levels of IL-1β and IL-18 by real-time PCR and ELISA, respectively. The results showed that the expression of both IL-1β and IL-18 was increased in ischemic muscles (Figure 5A-B), indicating the induction of pyroptosis in skeletal muscle. The increased cytokine levels were reduced by exosome treatment, and the effect was reversed by si-circHIPK3, suggesting that the ability of exosome to inhibit the expression of cytokines relies on circHIPK3 (Figure 5C-D). Real-time PCR and Western blot analysis showed that the mRNA and protein levels of NLRP3 and caspase-1 were increased several folds in ischemic muscles (Figure 5E-G). The expression of both NLRP3 and caspase-1 was significantly down regulated by treating the ischemic hindlimb with exosomes (Figure 5H-J). Taken together, these data suggest that exosomes can prevent skeletal muscle pyroptosis and the beneficial effect was mediated by circHIPK3.

### Exosomes stimulate the proliferation of C2C12 cells and inhibit NLRP3 and caspase-1 expression by releasing circHIPK3* in vitro*

**Figure 6:**
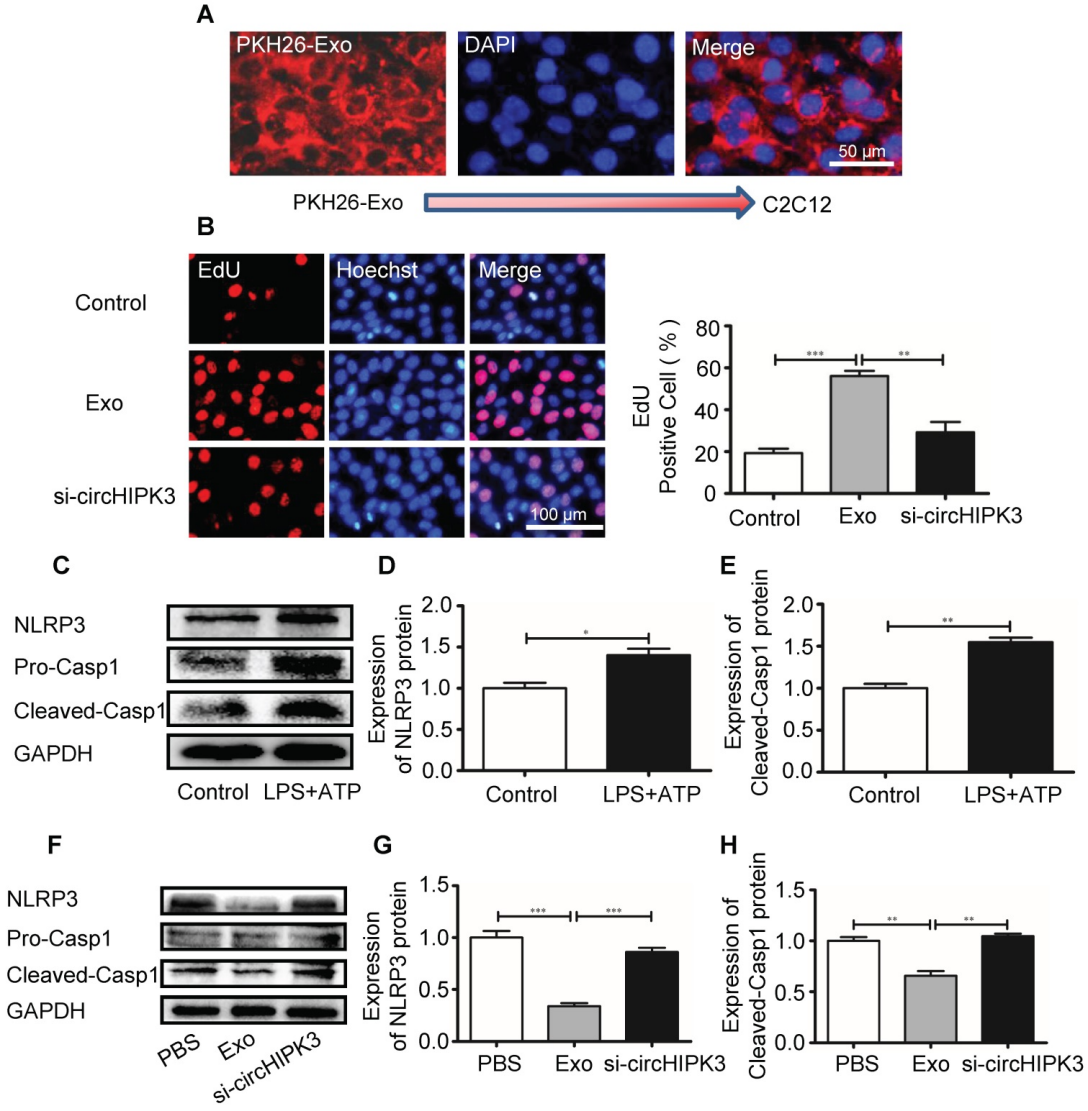
**circHIPK3 inhibits C2C12 cell pyroptosis in vitro. (A)** C2C12 cells were incubated with PKH26-labeled Exo for 24h and Exo uptake was detected by Fluorescence microscopy. Blue: nuclear staining (DAPI); Red: PKH-26-Exo staining (scale bar: 100 μm). **(B)** The proliferation of C2C12 cells was detected by EdU incorporation. The cells were pre-treated with Exo or Exo- si-circHIPK3. Blue: nuclear staining (Hoechst); Red: EdU staining (scale bar: 100 μm). **(C-E)** The protein levels of NLRP3 and Casp1 in C2C12 cells treated with LPS and ATP. **(F-H)** The protein levels of NLRP3 and Casp1of the PBS, Exo and Exo-si-circHIPK3 groups in LPS+ATP -treated C2C12 cells. Data is presented as mean ± standard deviation (n=3). *P <0.05, **P <0.01, ***P <0.001.

In order to delineate the mechanisms underlying circHIPK3-mediated tissue repair, we incubated the murine myoblast line C2C12 cells with exosomes. PKH26-labeled exosomes entered C2C12 cells as indicated by a red fluorescent signal (Figure 6A). EdU incorporation assay revealed that the numbers of proliferating cells were increased by exosome treatment (Figure 6B). We then investigated how exosomes affect the formation of inflammasome in C2C12 cells. C2C12 cells were pre-treated with LPS (100 ng/mL) and formation of inflammasome was triggered by adding ATP (2.5 mM) (Figure 6C-F). However, in the presence of exosome, NLRP3 and caspase-1 was significantly down regulated and the effect of exosomes was reversed by si-circHIPK3 (Figure 6G-I). These findings indicate that exosome can increase cell growth and prevent the formation of inflammasome by delivering circHIPK3.

### circHIPK3 serves as a sponge for miR-421

**Figure 7:**
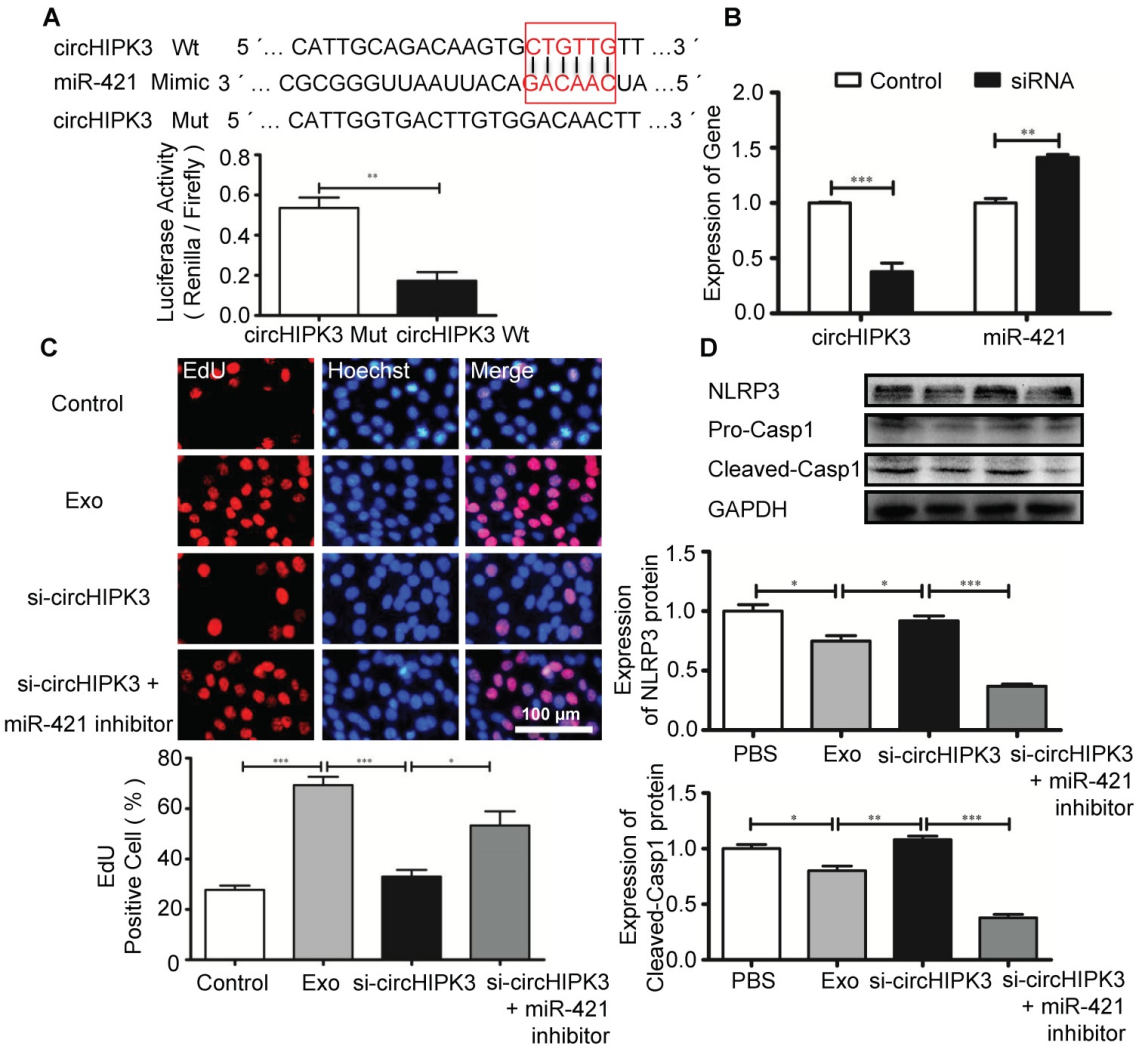
**circHIPK3 regulates the expression of miR-421. (A)** The bioinformatics analysis predicted the putative complementary sites within miR-421 and circHIPK3 (http://syslab5.nchu.edu.tw/CircNet/), and dual-Luciferase reporter assay showed that putative complementary sites within miR-421 with circHIPK3. **(B)** RT-PCR analysis showed that circHIPK3 silencing resulted in increased expression of miR-421 in C2C12 cells. **(C)** Immunofluorescence images showing the EdU positive cells in the control, Exo, Exo-si-circHIPK3, and Exo-si-circHIPK3+miR-421 inhibitor groups. Blue: nuclear staining (Hoechst); Red: EdU staining (scale bar: 100 μm). **(D)** NLRP3 and Casp1 protein levels in the PBS, Exo, Exo-si-circHIPK3, and Exo-si-circHIPK3+miR-421 inhibitor groups in LPS+ATP -treated C2C12 cells. Data is shown as mean ± standard deviation (n=3), *P <0.05, **P <0.01, ***P <0.001.

Circular RNAs can regulate gene expression by acting as miRNA sponges to reduce the number of freely available miRNA molecules 28. Bioinformatic analysis revealed that miR-421 contains binding sites for circHIPK3, and circRNA-miRNA interaction was confirmed by luciferase reporter assay (Figure 7A). Furthermore, circHIPK3 silencing in C2C12 cells resulted in increased expression of miR-421 (Figure 7B). EdU assay showed that Exo promoted cell proliferation, which was inhibited by si-circHIPK3 and the effect of circHIPK3 silencing was reversed by miR-421 inhibitor (Figure 7C). Western blot analysis revealed that the expression of NLRP3 and caspase-1 in C2C12 cells was decreased in the presence of the miR-421 inhibitor (Figure 7D). These data confirmed that miR-421 is a direct target of circHIPK3.

### FOXO3a is a direct target of miR-421

**Figure 8:**
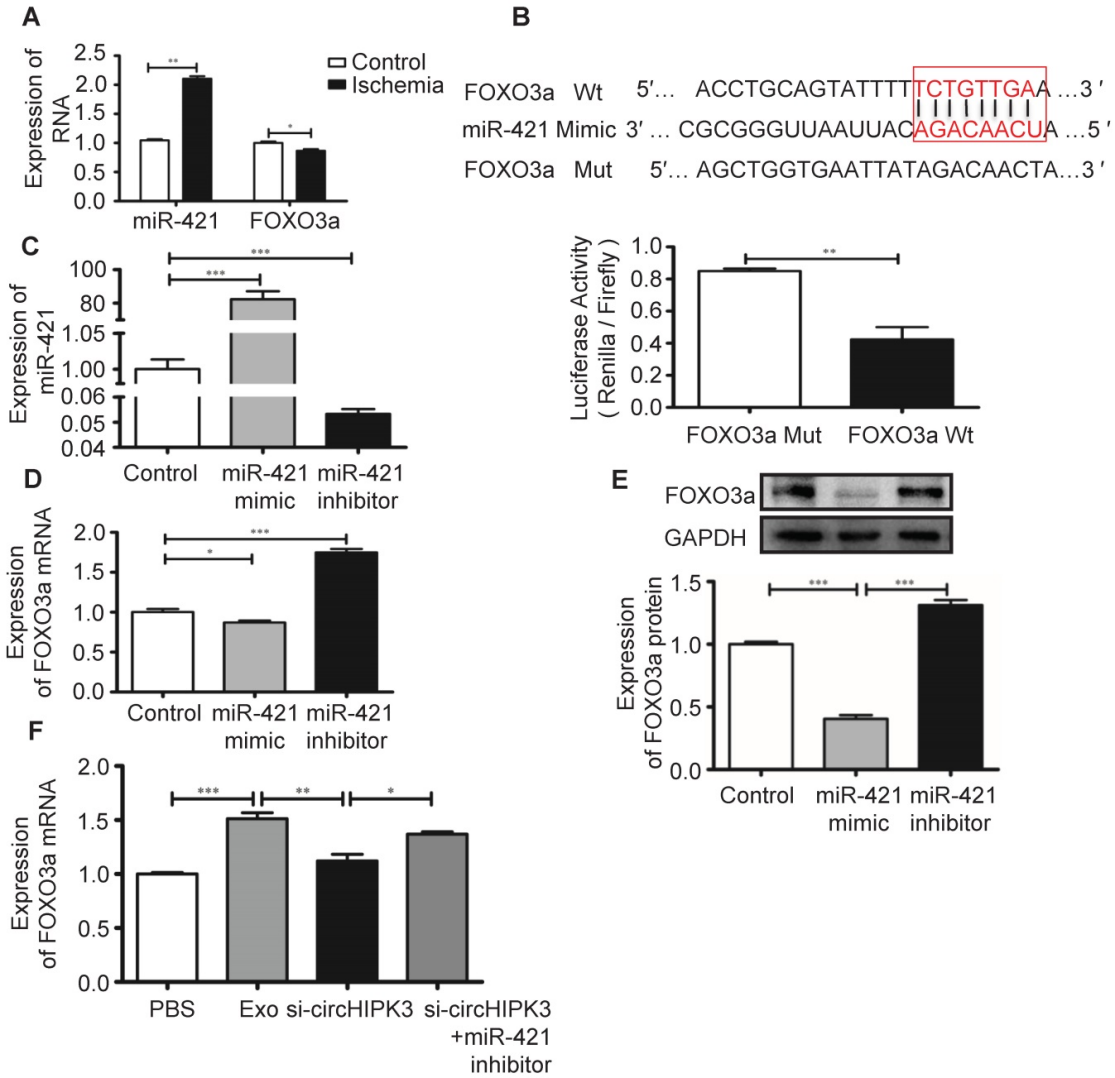
**FOXO3a is a downstream target of miR-421. (A)** RT-PCR analysis showed up regulation of miR-421 and down regulation of FOXO3a in ischemic muscle. **(B)** The bioinformatics analysis (starBase_V2.0) predicted the putative complementary sites within miR-421 and FOXO3a, and confirmed by dual-Luciferase reporter assay.** (C)** RT-PCR analysis of the levels of miR-421 in the control, miR-421 mimic and miR-421 inhibitor groups. **(D, E)** RT-PCR and Western blot analysis of the expression of FOXO3a treated by miR-421 mimic, or miR-421 inhibitor.** (F)** RT-PCR analysis of the levels of FOXO3a mRNA in the PBS, Exo, Exo-si-circHIPK3, and Exo-si-circHIPK3+miR-421 inhibitor groups in LPS+ATP -treated C2C12 cells. Data is presented as mean ± standard deviation (n=3), *P <0.05, **P <0.01, ***P <0.001.

FOXO3a is a transcription factor that regulates cell pyroptosis 12. In ischemic hindlimb tissue, increased miR-421 expression is associated with reduced mRNA expression of FOXO3a (Figure 8A). The potential binding sites between miR-421 and its target genes were predicted using bioinformatics analysis (starBase_V2.0). Results showed that FOXO3a has a good correlation with the miR-421 at 3'-UTR, which was confirmed by luciferase reporter assay (Figure 8B). We used miR-421 mimic or inhibitor to manipulate the expression of miR-421 in C2C12 cells (Figure 8C), and real-time PCR and Western blot analysis showed that the expression of FOXO3a was inhibited by miR-421 mimic but enhanced by miR-421 inhibitor (Figure 8D-E). Importantly, we showed that Exo can increase FOXO3a expression by delivering circHIPK3 because FOXO3a expression was inhibited by si-circHIPK3 but increased by miR-421 inhibitor (Figure 8F), which further confirmed that FOXO3a is a direct target of miR-421.

## Discussion

In this study, we found that circHIPK3 was down regulated in ischemic muscle of mouse hindlimbs. Using loss/gain-of function method, we demonstrated that FOXO3a is the direct target of miR-421, and that UMSC-Exo prevent ischemic injury by releasing circHIPK3, which in turn down regulate miR-421, resulting in increased expression of FOXO3a, leading to inhibition of pyroptosis and release of IL-1β and IL-18 (Figure S1).

Acute lower limb ischemia induces severe inflammatory reaction that leads to cell death 29-31. The molecular mechanisms underlying acute ischemic injury remain largely unknown, which limits the development of effective therapy 32. Emerging evidence suggests that exosome based therapies are effective in treating various diseases including myocardial infarction 13, 33-35. As a shuttle vehicle between cells, exosomes transport functional molecules such as miRNA and circRNA to regulate gene expression in recipient cells 36. It has been shown that both miRNA and lncRNA are involved in tissue repair and regeneration 24, 37. However, reports on the role of exosome derived circRNA in repairing ischemic injury are scarce. Our present study for the first time demonstrated that exosome prevented ischemia-induced pyroptosis in mouse hindlimb by delivering circHIPK3. Previous studies showed that circHIPK3 was mainly involved in the regulation of cell proliferation, autophagy and other biological processes 38-40. However, we found that circHIPK3 may also be involved in the regulation of pyroptosis.

Pyroptosis is a type of programmed cell death resulting from inflammation 2. Unlike apoptosis, pyroptosis is a caspase-1 dependent process that leads to rapid cell lysis and the release of inflammatory content that disturbing neighboring cells 3. It is known that acute ischemic injury of lower limbs is accompanied by the release of pro-inflammatory cytokines and activation of inflammasome NLRP3, which induces pyroptosis 4, 5. Once activated, NLRP3 triggers pyroptosis by releasing inflammatory cytokines including IL-1β 6, 7. Recent studies showed that myocardial infarction induced cardiac dysfunction can be effectively improved by inhibiting pyroptosis 41, 42. We now provide new evidence that the expression of both inflammasome NLRP3 and caspase-1 was significantly down regulated by treating the ischemic hindlimb with exosomes, and this effect was reversed by si-circHIPK3. These data suggest that the inhibitory effect of exosomes on the activation of NLRP3 and caspase-1 is mediated by circHIPK3. These findings provide a potential therapeutic approach to prevent the activation of inflammasome and pyroptosis.

FOXO3a is a transcription factor of the O subclass of the forkhead family, and is conserved among various species 12. It has been shown that FOXO3a can inhibit pyroptosis by regulating inflammatory response 11, 12. High glucose induced pyroptosis in cardiomyocytes is mediated by miR-30 which in turn inactivate FOXO3a 12. In tumor associated myeloid cells, FOXO3a inhibits inflammatory signaling by preventing NF-κB nuclear translocation 11. After ischemic injury in mice, pyroptosis can occur within 1 hour, but apoptosis usually occurs after 12 hours 43. Therefore, inhibition of pyroptosis is more important and requires early intervention. This study provides a novel effective approach that UMSC-Exo prevents pyroptosis by releasing circHIPK3, which in turn down regulate miR-421, resulting in increased expression of FOXO3a.

In conclusion, our study demonstrates that UMSC-Exo enhance ischemic hindlimb repair by delivering circHIPK3 to inhibit pyroptosis in skeletal muscle cells. We provided evidence that circHIPK3 serves as a miR-421 sponge to inhibit inflammation, increases FOXO3a expression and prevents the activation of inflammasome NLRP3 and caspase-1. Therefore, our findings not only shed new light into the mechanisms of exosome/circHIPK3 based therapy, but also provided an effective therapeutic approach for treating ischemic injury.

## Supplementary Material

Supplementary figures and tables.
