# Supplementary material for: Stem cell-derived exosomes prevent pyroptosis and repair ischemic muscle injury through a novel exosome/circHIPK3/ FOXO3a pathway
Source: Theranostics. 2020 May 18;10(15):6728–42. doi: 10.7150/thno.42259 (PMC7295049; doi:10.7150/thno.42259)
Supplement: Supplementary file 1 — Supplementary figures and tables. [file thnov10p6728s1.pdf]

Supplemental File

Stem cell-derived exosomes prevent pyroptosis and repair ischemic muscle injury through a novel exosome/circHIPK3/ FOXO3a pathway

Bing Yan[a#], Yu Zhang[a#], Chun Liang[b#], Bin Liu[c#], Fengzhi Ding[a], Yanli Wang[a], Bao Zhu[a], Ranzun Zhao[d], Xi-Yong Yu[e], Yangxin Li[a,*]

Supplemental Figure 1

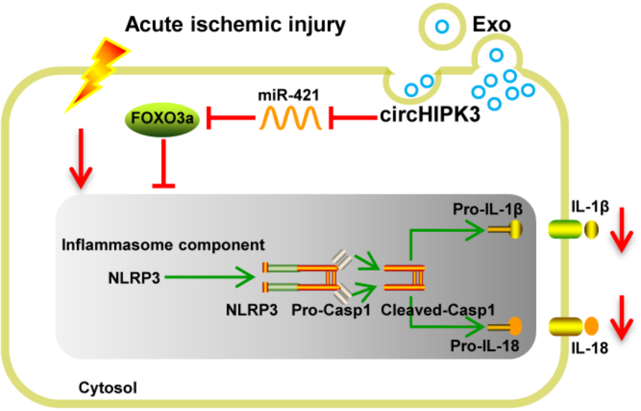

A schematic illustration to demonstrate the mechanisms underlying exosome's therapeutic effect.

Supplemental Figure 2

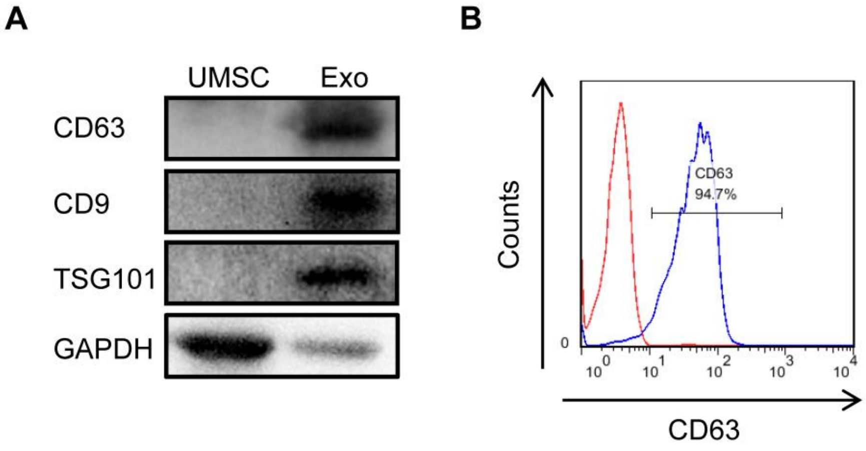

The characterization of exosomes. (A) The exosomal markers CD63, CD9 and TSG101 in UMSC cells and exosomes were analyzed byWestern blot. (B) The

exosomal surface marker CD63 was analyzed by flow cytometry.

Supplemental Figure 3

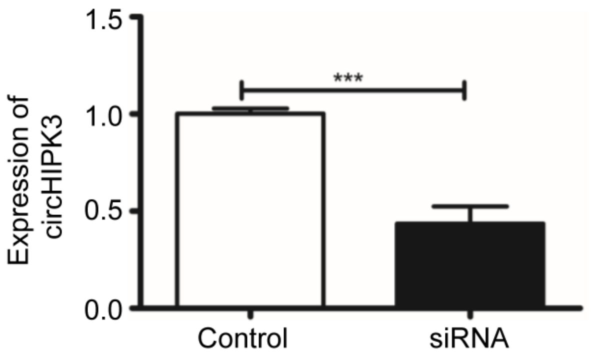

The knockdown efficiency of circHIPK3. The expression of circHIPK3 was analyzed in control and siRNA exosome groups by RT-PCR， ***P <0.001.
